# Design, Synthesis and Evaluation of Hesperetin Derivatives as Potential Multifunctional Anti-Alzheimer Agents

**DOI:** 10.3390/molecules22071067

**Published:** 2017-06-26

**Authors:** Bo Li, Ai-Ling Huang, Yi-Long Zhang, Zeng Li, Hai-Wen Ding, Cheng Huang, Xiao-Ming Meng, Jun Li

**Affiliations:** 1 Anhui Province Key Laboratory of Major Autoimmune Diseases, Anhui Institute of Innovative Drugs, School of Pharmacy, Anhui Medical University, 230000 Hefei, China; AHlibo6812@163.com (B.L.); 131180308002@163.com (A.-L.H.); ahmu_zhangyl@126.com(Y.-L.Z.); lizeng@ahmu.edu.cn (Z.L.); 17730227009@163.com (H.-W.D.); huangcheng812@ahmu.edu.cn (C.H.); mengxiaomingahmu@163.com (X.-M.M.); 2 The Key Laboratory of Anti-Inflammatory and Immune Medicines, Ministry of Education, 230000 Hefei, China; 3 Institute for Liver Diseases, Anhui Medical University, 230000 Hefei, China

**Keywords:** hesperetin derivatives, acetylcholinesterase (AChE), butyrylcholine esterase (BuChE), β-amyloid (Aβ), multifunctional

## Abstract

In this study we designed and synthesized a series of new hesperetin derivatives on the basis of the structural characteristics of acetylcholinesterase (AChE) dual-site inhibitors. The activity of the novel derivatives was also evaluated. Results showed that the synthesized hesperetin derivatives displayed stronger inhibitory activity against AChE and higher selectivity than butyrylcholine esterase (BuChE) (selectivity index values from 68 to 305). The Lineweaver-Burk plot and molecular docking study showed that these compounds targeted both the peripheral anionic site (PAS) and catalytic active site (CAS) of AChE. The derivatives also showed a potent self-induced β-amyloid (Aβ) aggregation inhibition and a peroxyl radical absorbance activity. Moreover, compound **4f** significantly protected PC12 neurons against H_2_O_2_-induced cell death at low concentrations. Cytotoxicity assay showed that the low concentration of the derivatives does not affect the viability of the SH-SY5Y neurons. Thus, these hesperetin derivatives are potential multifunctional agents for further development for the treatment of Alzheimer’s disease.

## 1. Introduction

Alzheimer’s disease (AD) is a chronic neurodegenerative brain disorder that is characterized by cognitive impairment, memory loss, and dementia [1]. According to one report, 36 million people in the world were living with dementia in 2010, and the number will double every 20 years, eventually leading to more than 115 million people with AD in 2050 [2]. The pathogenesis of AD remains unknown, although many hypotheses have been developed. Among them, brain cholinergic neuron damage, amyloid-β (Aβ) cascade, and oxidative stress hypotheses are widely recognized and are speculated to be the dominant causes of AD pathogenesis [3,4,5].

According to the cholinergic hypothesis, acetylcholinesterase (AChE) inhibitors, such as tacrine, donepezil, rivastigmine, and galantamine, are commonly used in clinical practice to treat AD [6]. However, AChE inhibitors are not ideal for the treatment of AD. They can improve a patient’s neuropsychiatric symptoms, but cannot prevent or slow down AD development. The latest research has shown that AChE possibly plays multiple functions in AD development because its peripheral anionic site (PAS) can accelerate the production of Aβ protein and promote its aggregation [7]. Therefore, the catalytic active site (CAS) and the PAS dual-binding site inhibitor that act on AChE may exhibit multiple targets of action for AD treatment. A simple way of designing the dual-site AChE inhibitor is to connect two pharmacophores to a dyad in a certain manner. This dimer can simultaneously act on the CAS and the PAS of AChE to improve the inhibitory activity on AChE and inhibit AChE-induced Aβ aggregation [7,8]. This finding has become the new basis for developing new cholinesterase inhibitors. AChE and BuChE both exist in the body of mammals. There is clinical evidence that suggests BuChE may compensate for the lack of AChE [9]. However, BuChE mainly exists in the peripheral nervous system. Thus, BuChE inhibition causes side effects on the peripheral nervous system [10]. The dose effect curves of tremor (central effect) and salivation (peripheral effect) prove that the highly selective cholinesterase inhibitor donepezil has a better therapeutic index inhibition than tacrine, which can inhibit both BuChE and AChE [11,12]. Moreover, recent reports showed that the activity of BuChE decreased in the nerve synapses of AD patients with the use of donepezil [13]. These results indicate that AChE inhibitors that exhibit high selectivity and high activity have excellent development prospects in the research and development of AD drugs.

**Scheme 1:**
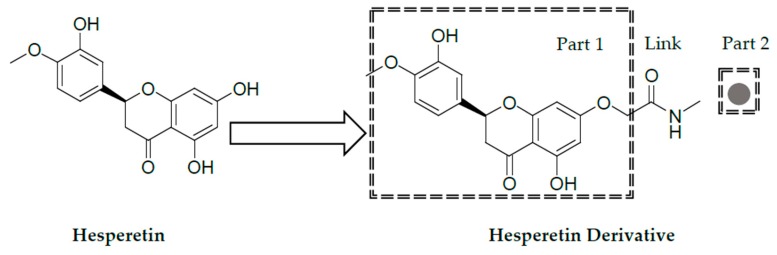
Structure of hesperetin and the design idea of dual-target AChE inhibitors.

In Chinese Traditional Medicine citrus is the fruit skin of the Rutaceae plants that produce oranges (*Citrus reticulata Blanco*) and its cultivars. Hesperitin (hesperetin 7-*O*-glucoside) and hesperidin (hesperidin 7-*O*-rutinoside) are the primary active contents of citrus fruits that have diverse pharmacological activities, such as antioxidant, anti-inflammatory, antibacterial, anti-cancer, anti-hepatic fibrosis, and immunity adjustment properties [14,15,16,17,18,19,20,21]. In our early efforts to identify novel AChE inhibitors, hesperitin was identified as an AChE inhibitor with an IC_50_ value of 1.02 μM. On the basis of the dual-site strategy that was designed according to the structural characteristics of hesperitin and the benzene or heteroaromatic ring side chain structure of AChE inhibitors [22], we designed a dual-site AChE inhibitor that acts on both the AChE central CAS and PAS with the benzene or heteroaromatic ring terminal side chain structure as the parent (Scheme 1). We wanted to synthesize the hesperitin derivatives where part 1 can binds to the PAS and part 2 can bind to the CAS. We aimed to improve the AChE inhibitory activity as well as the AChE and BuChE selectivity, by introducing a benzene or heteroaromatic ring terminal side chain. Furthermore, these derivatives showed potent antioxidant activities and excellent binding affinities with Aβ in vitro binding assay.

## 2. Results and Discussion

### 2.1. Chemistry

**Scheme 2:**
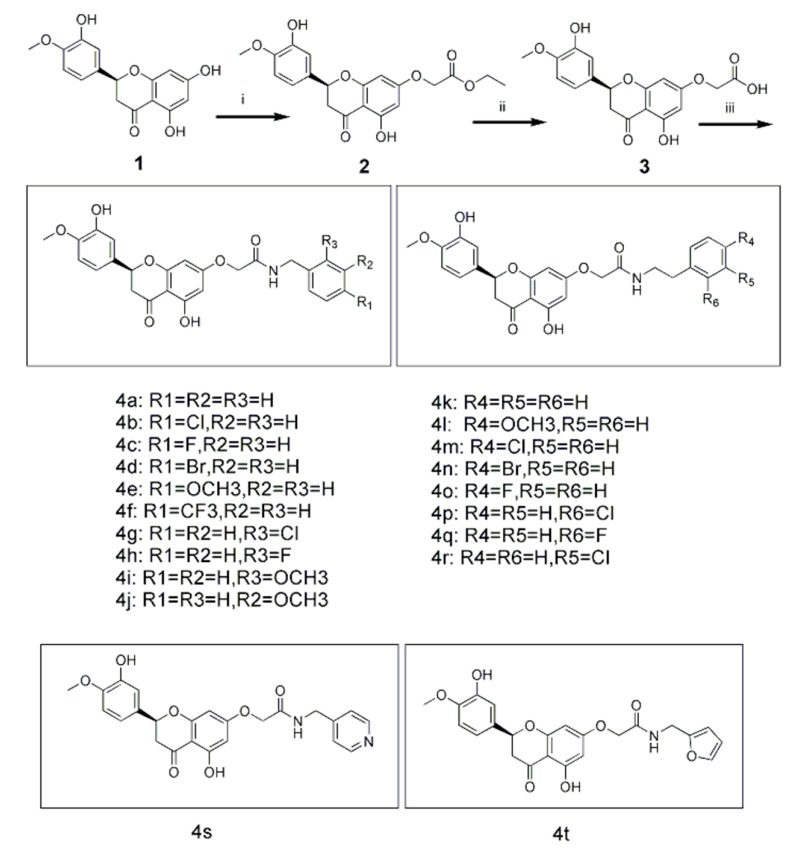
Synthesis of hesperetin derivatives. *Reagents and conditions*: (i) ethyl bromoacetate, K_2_CO_3_, I_2_, acetone, reflux; (ii) 5% Na_2_CO_3_, DMSO, 90 °C, reflux, 10% HCl; (iii) EDC, HOBT, DMF, CHCl_3_, RNH_2_, r.t.

The synthetic route to the target compounds **4a**–**4t** that starts from commercially available hesperetin (**1**) is shown in Scheme 2. The reaction of **1** with ethyl bromoacetate in ethanol provided **2** in 82% yield. Then, treatment of **2** with excess sodium carbonate in ethanol and H_2_O, followed by acidification with aqueous HCl, resulted in intermediate **3** in 75% yield. Finally, the reaction of **3** with different amines produced targets **4a**–**4t** in 40–72% yields. The structures of the target compounds were confirmed by ^1^H-NMR, ^13^C-NMR, and MS.

### 2.2. Inhibitory Study of Hesperetin Derivatives on AChE and BuChE

The inhibitory activity of the derivatives was evaluated against AChE and BuChE with tacrine as the positive control using the method of Ellman et al. [23]. The IC_50_ values and the selectivity index for the inhibition of AChE and BuChE were summarized in Table 1.

Table 1 shows that all the derivatives, except for the parent compound hesperetin, exhibited strong inhibitory ability and selectivity for AChE after the introduction of a benzene ring or heteroaromatic ring terminal side chain. The selective index ranged from 68 to 305, and the IC_50_ values were shown in nanomole concentration level. The inhibitory activities of these derivatives were better than those of tacrine. On the one hand, we speculated that these side chains were similar to the structure of the acetylcholine molecule, which easily enters the enzyme activity pocket and interacts with the amino acid residue in the triplet catalytic center. On the other hand, the side chain can combine with the active site of the enzyme and guide the molecules into the narrow active valley of cholinesterase such that the other groups mutually combine with the amino acid residues in the PAS region. BuChE contains no PAS binding site and its active center is wider than that of AChE. Therefore, the introduction of the side chain exerted a lesser effect on the inhibitory activity against BuChE than AChE.

Changes in the side chains can also significantly affect the inhibitory activity of the hesperetin derivatives on cholinesterase. In the comparison of the side chains on the benzene ring at different locations and different electronegative substituents, such as hydrogen atoms, methoxy group, fluorine atom, chlorine atom, bromine atom, and trifluoromethyl group, compound **4f** showed the best inhibitory activity. This may be because the electronegative group is located at the *para* position of the benzene ring, and the electronegativity of the substituent is greater, so the compound has the best inhibitory activity.

### 2.3. Kinetic Studies for the Inhibition of AChE

**Figure 1:**
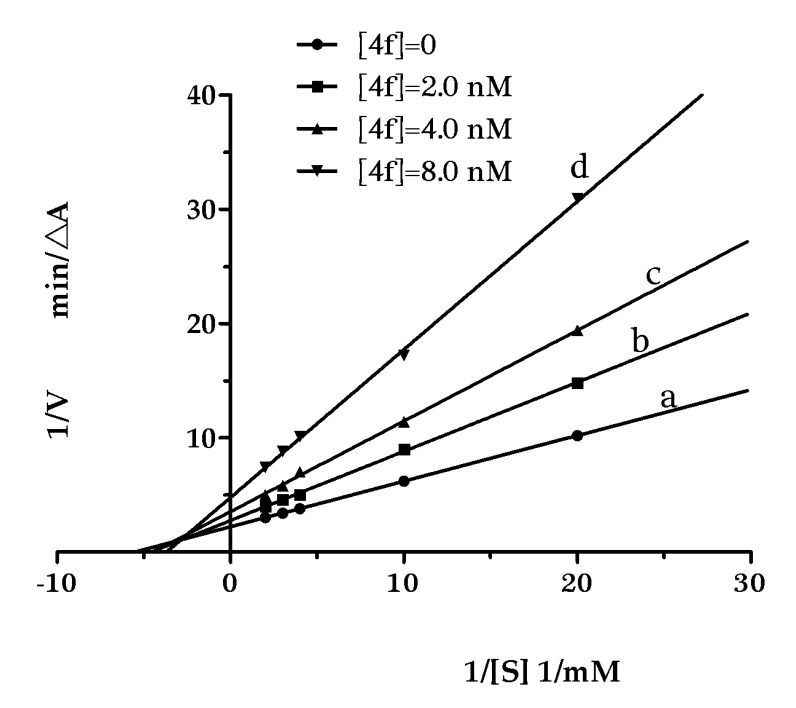
Lineweaver-Burk plot for the inhibition of AChE by **4f**.

To identify the inhibition type of hesperetin derivatives against AChE, a kinetic study was performed using compound **4f**, and the steady-state inhibition data for AChE are shown in Figure 1. The Lineweaver–Burk plots showed that all straight lines were intersected in the second quadranta and Ki ^a^ = 5.51, Ki ^b^ = 4.67, Ki ^c^ = 4.27, Ki ^d^ = 3.28, which characterizes a typical mixed-type inhibition [24]. Therefore, we speculated that the synthesized hesperetin derivatives interacted with two AChE functional sites, CAS and PAS. Thus, the derivatives showed a strong inhibitory effect that was consistent with the molecular docking experiment.

### 2.4. Study on the Interaction between ***4f*** and AChE by Molecular Docking Method

**Figure 2:**
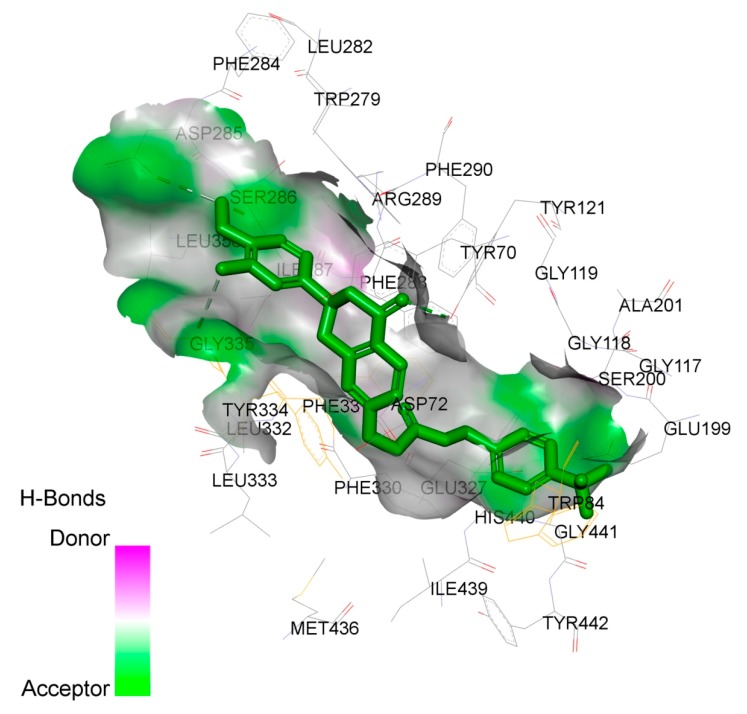
Docking models of compound-enzyme complex: (**4f**)-TcAChE complex.

In recent years, molecular docking simulations have become an important tool for understanding the interaction mode and the structure-activity relationships of ligands with receptors. To obtain useful information about the binding interactions between the hesperetin derivatives and AChE, molecular docking simulations were performed using the AutoDock4.0 package with the PyMOL program, and the result is shown in Figure 2. Figure 2 shows that the optimal structural conformation of the ligand molecule **4f** was based on the docking energy value during the docking between **4f** and cholinesterase. The docking result showed that the ligand molecule interacted with the functional groups of the amino acid residues found along the active valley of the AChE (Figure 2). The parent structure of **4f** was bound to the PAS region and displayed a classic π-π stacking interaction between Tyr334, the 2-phenyl of **4f**, and Phe331, with a ring-to-ring distance of 3.4 and 3.1 Å. The carbonyl group of the hesperetin moiety established a hydrogen bond with the hydroxyl group of the main chain of TRR121. The hydroxyl group and methoxyl group in the 2-substituted benzene ring of the parent structure established a hydrogen bond with ASP285 and GLY335 respectively. Furthermore, the conformation of the amide group side chain matched the enzyme active valley well. The benzene ring in the terminal was bound to the CAS with a classic π-π stacking interaction with TRP84. The ring-to-ring distance was 3.6 Å. These results showed that compound **4f** could react with both the PAS and the CAS of the enzyme, thus resulting in the strong AChE inhibitory effect.

### 2.5. Antioxidant Activity

Reactive oxygen species (ROS) have been identified as important mediators of cell structure damage to proteins, lipids, and nucleic acids. ROS have been associated with AD, aging, and other neurodegenerative disorders [25]. In normal physiological conditions, the host antioxidant defenses control the level of oxygen free radicals. However, when free radicals have overwhelmed these defenses, cellular damage occurs [26]. Thus, an antioxidant might be a therapeutic strategy to prevent the development of AD.

The antioxidant activities of all synthesized hesperetin derivatives were evaluated by following the oxygen radical absorbance capacity by the fluorescein (ORAC-FL) method and the results are shown in Table 1 [27]. Vitamin E analogue Trolox was used as the standard, and the results were expressed as Trolox equivalents. Hesperetin, which has an ORAC-FL value of 5.1 Trolox equivalents, was also tested. All the tested derivatives exhibited potent peroxyl radical absorbance capacities that ranged from 2.4- to 3.2-fold that of Trolox. As expected, the introduction of the side chain in the hesperetin parent decreased the radical capturing capacity. These results indicate that the phenolic hydroxy in the hesperetin was responsible for the radical scavenging ability. However, the compounds still demonstrated excellent antioxidant activity. Compound **4a** was the strongest antioxidant (ORAC value = 3.2) among the other compounds. Compounds **4f**, **4g** and **4o** showed slightly lower activities (ORAC values = 3.0, 3.1, and 3.1, respectively).

### 2.6. Inhibition of Self-Mediated Aβ (1–42) Aggregation

**Figure 3:**
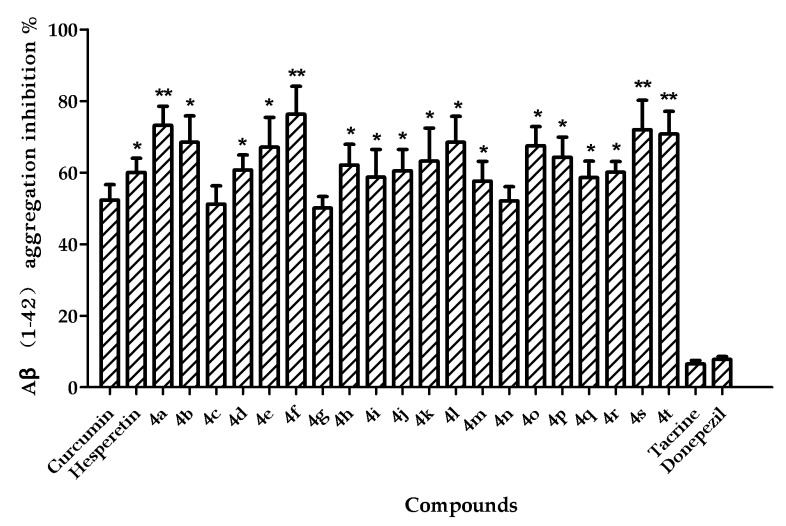
Effects of the compounds on Aβ (1–42) peptide aggregation inhibition. The compounds were used at a concentration of 20 μM. (* *P* < 0.05 and ** *P* < 0.01 vs. curcumin group).

Aβ (1–40) and Aβ (1–42) are the two types of Aβ peptides found in amyloid plaques. They originated from the enzymatic cleavage of the amyloid precursor protein operated by β- and γ-secretases. Aβ (1–42) is significantly more fibrillogenic than Aβ (1–40). Therefore, Aβ (1–42) was selected to study the inhibitory activity of the hesperetin derivatives [28]. The activity of the derivatives was assessed by thioflavin T fluorescence assay using curcumin as the standard. As shown in Figure 3, most of the tested compounds exhibited distinctly higher inhibitory activity than curcumin. Compounds **4a**, **4f**, **4s** and **4t** showed the most potent inhibitory activities with 73.2%, 76.3%, 72.0% and 70.8% inhibition rates, respectively. Furthermore, the IC_50_ values of compound (**4f**) and curcumin were measured, compound **4f** (IC_50_ = 8.47 ± 0.44 μM) was approximately two-fold more potent than curcumin (IC_50_ = 16.25 ± 0.15 μM). The hypothesis was that organic compounds with antioxidant activity could specifically bind to Aβ or Aβ fibril; however, the mechanisms are yet unknown [29].

### 2.7. Neuroprotective Effect Against H_2_O_2_-Induced Cell Death in PC12 Neurons

**Figure 4:**
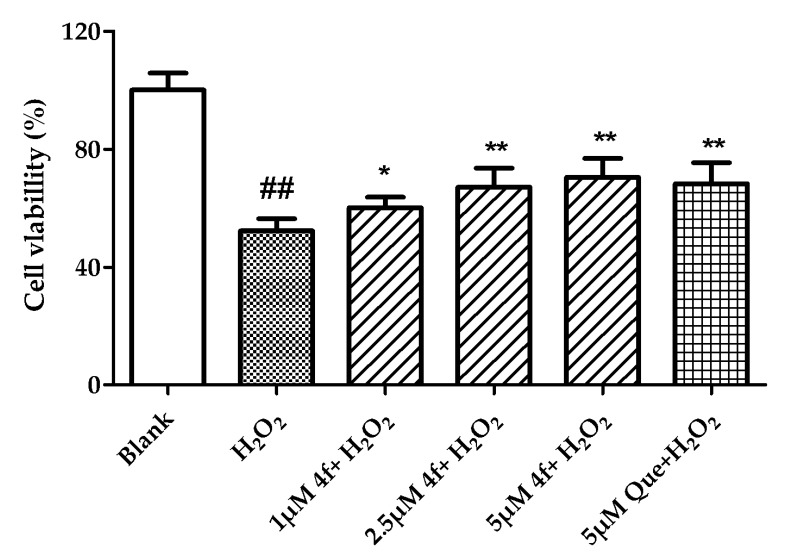
Neuroprotective activity of compound **4f** against H_2_O_2_-induced cell death in PC12 cells. (^##^ *P* < 0.01 vs. Blank group. * *P* < 0.05 and ** *P* < 0.01 vs. H_2_O_2_-treated group.).

The neuroprotective activity of compound **4f** against oxidative stress-induced cell death in PC12 cells was evaluated. The obtained data are shown in Figure 4. Compound **4f** was evaluated at concentrations of 1, 2.5 and 5 µM, and quercetin was used as the reference compound. Compound **4f** exhibited no cytotoxicity toward the PC12 cells at the tested concentrations. H_2_O_2_ (200 µM) reduced the cell viability to 50.7% compared to the control. The pretreatment of PC12 cells with compound **4f** significantly protected the neurons against cell death in all the concentrations (*p*-value < 0.01). Compound **4f** showed a slightly better protective capability than quercetin at a concentration of 5 µM. These observations indicated that compound **4f** has the potential to be an excellent multifunctional agent for the treatment of AD.

### 2.8. Effects of Hesperetin Derivatives on Neurocyte Viability

As potential anti-AD agents, hesperetin derivatives must exhibit low toxicity. Thus, we used the methylthiazolyl tetrazolium (MTT) method [30] to perform a neurocyte (SH-SY5Y) toxicity test in vitro. The results in Table 2 showed that the incubation of the SH-SY5Y cell strain with 1µM and 10 µM concentrations of all compounds for 48 h did not influence the cell viability of the strain significantly. Furthermore, the neurocyte inhibition rate was below 10%.

When the concentration was increased to 50 µM and 100 µM, the compounds exhibited certain cytotoxicity. However, compared with the cholinesterase inhibitor’s activity at the nanomole level, the target compounds exhibited a wide therapeutic safety range. Cholinesterase could also be inhibited under 100 nmol or lower concentrations to prevent severe toxicity to the body.

## 3. Materials and Methods

### 3.1. General Information

^1^H-NMR spectra were recorded using TMS as the internal standard in CDCl_3_ or dimethyl sulfoxide (DMSO-*d*_6_) solutions on a Bruker 400 MHz instrument (Bruker, Karlsruhe, Germany). MS data were obtained by using a LC-MS-2010A spectrometer (Shimadzu, Kyoto, Japan) with an ESI or ACPI mass selective detector. Elemental analysis was measured by an Elementar Vario EL CHNS Elemental Analyzer (Elementar, Hanau, Germany), and data were uncorrected.

### 3.2. Chemistry

#### 3.2.1. Synthesis of Intermediate **2**

Hesperetin (3.02 g, 10 mmol), ethyl 2-bromoacetate (1.66 g, 15 mmol), anhydrous K_2_CO_3_ (2.76 g, 20 mmol) and catalytic amount KI were added in anhydrous ethanol (150 mL), the mixture was refluxed for 1 h. The solution was evaporated, and the residue was poured into water and extracted with ethyl acetate. The combined organic layers were dried over anhydrous Na_2_SO_4_, and then concentrated to give white solid **2**, which was used without further purification. Yield 2.82 g (70%).

#### 3.2.2. Synthesis of Intermediate **3**

Compound **2** (2.82 g, 7.1 mmol), 5% Na_2_CO_3_ (10 mL), and ethanol (40 mL) were stirred at 50 °C for 8 h. Then the reaction mixture was poured into 10% HCl (200 mL), and filtered. The filter cake was recrystallized from EtOH to give yellow solid 2.02 g; Yield 80%, ^1^H-NMR (400 MHz, DMSO-*d*_6_) δ 12.48 (s, 1H, COOH), 12.10 (s, 1H, 5-OH), 9.11 (s, 1H, 3′-OH), 6.90 (dt, *J* = 9.6, 4.7 Hz, 3H, 2′-H, 3′-H, 5′-H), 6.15 (d, *J* = 2.1 Hz, 1H, 8-H), 6.13 (d, *J* = 2.1 Hz, 1H, 6-H), 5.49 (dd, *J* = 12.2, 2.5 Hz, 1H, 2-H), 4.36 (s, 2H, C=OCH_2_O), 3.26 (dd, *J* = 17.2, 12.0 Hz, 1H, 3-H), 2.75 (dd, *J* = 17.1, 2.8 Hz, 1H, 3-H). ^13^C-NMR (101 MHz, DMSO-*d*_6_) δ 196.83, 169.40, 165.80, 163.03, 162.65, 147.93, 146.46, 130.92, 117.71, 114.08, 111.96, 102.88, 95.12, 94.23, 78.44, 64.68, 55.65, 42.12. TOF-HRMS: *m*/*z* [M + H]^+^ calcd. for C_18_H_17_N_2_O_3_: 309.1234; found: 309.1233.

#### 3.2.3. General Procedures for the Preparation of Target Compounds **4a**–**4t**

Compound **3** (0.37 g, 1 mmol), 1-[3-(dimethylamino)propyl]-3-ethylcarbodiimide (EDC, 0.29 g, 1.5 mmol), 1-hydroxybenzotrizole (HOBT, 0.20 g, 1.5 mmol) and CHCl_3_ (20 mL) were stirred at room temperature for 1 h, and then different amines (3 mmol) were added. The solution was stirred at room temperature overnight. Then the solvent was poured into water and extracted with ethyl acetate (20 mL × 3). The solution was dried over anhydrous Na_2_SO_4_ and concentrated. The residues were purified by flash chromatography with chloroform/petroleum ether (2:1, *v*/*v*) elution.

*N-Benzyl-2-[5-hydroxy-2-(3-hydroxy-4-methoxyphenyl)-4-oxochroman-7-yloxy]-acetamide* (**4a**). White solid (61% yield); ^1^H-NMR (400 MHz, CDCl_3_) δ 11.97 (s, 1H, 5-OH), 7.37–7.27 (m, 5H, Ar-H), 7.02 (d, *J* = 2.0 Hz, 1H, 2′-H), 6.92 (dd, *J* = 8.4, 1.9 Hz, 1H, 5′-H), 6.88 (d, *J* = 8.3 Hz, 1H, 4′-H), 6.74 (t, *J* = 5.0 Hz, 1H, NH), 6.07 (d, *J* = 2.4 Hz, 1H, 8-H), 6.05 (d, *J* = 2.4 Hz, 1H, 6-H), 5.33 (dd, *J* = 12.8, 3.1 Hz, 1H, 2-H), 4.54 (d, *J* = 5.1 Hz, 2H, NCH_2_), 4.54(s, 2H, C=OCH_2_O), 3.92 (s, 3H, OCH_3_), 3.08 (dd, *J* = 17.2, 12.8 Hz, 1H, 3-H), 2.81 (dd, *J* = 17.2, 3.1 Hz, 1H, 3-H). APCI-MS: calculated for [M + Na]^+^ (C_25_H_23_NNaO_7_) requires *m*/*z* 472.1367, found 472.1363.

*N-(4-Chlorobenzyl)-2-[5-hydroxy-2-(3-hydroxy-4-methoxyphenyl)-4-oxochroman-7-yloxy]-acetamide* (**4b**). White solid (58% yield); ^1^H-NMR (400 MHz, CDCl_3_) δ 11.98 (s, 5-OH), 7.33–7.28 (m, 2H, Ar-H), 7.25–7.20 (m, 2H, Ar-H), 7.03 (d, *J* = 2.0 Hz, 1H, 2′-H), 6.92 (dd, *J* = 8.4, 1.9 Hz, 1H, 5′-H), 6.88 (d, *J* = 8.3 Hz, 1H, 4′-H), 6.74 (t, *J* = 5.7 Hz, 1H, NH), 6.07 (d, *J* = 2.4 Hz, 1H, 8-H), 6.04 (d, *J* = 2.4 Hz, 1H, 6-H), 5.34 (dd, *J* = 12.8, 3.1 Hz, 1H, 2-H), 4.53 (s, 2H, C=OCH_2_O), 4.50 (d, *J* = 6.1 Hz, 2H, NCH_2_), 3.92 (s, 3H, OCH_3_), 3.09 (dd, *J* = 17.2, 12.8 Hz, 1H, 3-H), 2.82 (dd, *J* = 17.2, 3.1 Hz, 1H, 3-H). APCI-MS: calculated for [M + Na]^+^ (C_25_H_22_ClNNaO_7_) requires *m*/*z* 506.0977, found 506.0995.

*N-(4-Fluorobenzyl)-2-[5-hydroxy-2-(3-hydroxy-4-methoxyphenyl)-4-oxochroman-7-yloxy]-acetamide* (**4c**). White solid (63% yield); ^1^H-NMR (400 MHz, CDCl_3_) δ 11.98 (s, 1H, 5-OH), 7.48–7.43 (m, 2H, Ar-H), 7.16 (d, *J* = 8.3 Hz, 2H, Ar-H), 7.03 (d, *J* = 1.9 Hz, 1H, 2′-H), 6.92 (dd, *J* = 8.3, 2.0 Hz, 1H, 5′-H), 6.88 (d, *J* = 8.3 Hz, 1H, 4′-H), 6.76 (t, *J* = 5.6 Hz, 1H, NH), 6.07 (d, *J* = 2.3 Hz, 1H, 8-H), 6.04 (d, *J* = 2.2 Hz, 1H, 6-H), 5.33 (dd, *J* = 12.8, 3.0 Hz, 1H, 2-H), 4.53 (s, 2H, C=OCH_2_O), 4.48 (d, *J* = 6.1 Hz, 2H, NCH_2_), 3.92 (s, 3H, OCH_3_), 3.08 (dd, *J* = 17.2, 12.8 Hz, 1H, 3-H), 2.81 (dd, *J* = 17.2, 3.1 Hz, 1H, 3-H). APCI-MS: calculated for [M + Na]^+^ (C_25_H_22_FNNaO_7_) requires *m*/*z* 490.1273, found 490.1272.

*N-(4-Bromobenzyl)-2-[5-hydroxy-2-(3-hydroxy-4-methoxyphenyl)-4-oxochroman-7-yloxy]-acetamide* (**4d**). White solid (65% yield); ^1^H-NMR (400 MHz, CDCl_3_) δ 11.98 (s, 1H, 5-OH), 7.48–7.43 (m, 2H, Ar-H), 7.16 (d, *J* = 8.3 Hz, 2H, Ar-H), 7.03 (d, *J* = 1.9 Hz, 1H, 2′-H), 6.92 (dd, *J* = 8.3, 2.0 Hz, 1H, 5′-H), 6.88 (d, *J* = 8.3 Hz, 1H, 4′-H), 6.76 (t, *J* = 5.6 Hz, 1H, NH), 6.07 (d, *J* = 2.3 Hz, 1H, 8-H), 6.04 (d, *J* = 2.2 Hz, 1H, 6-H), 5.33 (dd, *J* = 12.8, 3.0 Hz, 1H, 2-H), 4.53 (s, 2H, C=OCH_2_O), 4.48 (d, *J* = 6.1 Hz, 2H, NCH_2_), 3.92 (s, 3H, OCH_3_), 3.08 (dd, *J* = 17.2, 12.8 Hz, 1H, 3-H), 2.81 (dd, *J* = 17.2, 3.1 Hz, 1H, 3-H). APCI-MS: calculated for [M + Na]^+^ (C_25_H_22_BrNNaO_7_) requires *m*/*z* 550.0413, found 550.0412.

*2-[5-Hydroxy-2-(3-hydroxy-4-methoxyphenyl)-4-oxochroman-7-yloxy]-N-(4-methoxy-benzyl)acetamide* (**4e**). White solid (58% yield); ^1^H-NMR (400 MHz, CDCl_3_) δ 11.97 (s, 1H, 5-OH), 7.24–7.19 (m, 2H, Ar-H), 7.02 (d, *J* = 1.9 Hz, 1H, 2′-H), 6.91 (dd, *J* = 8.3, 1.9 Hz, 1H, 5′-H), 6.88 (d, *J* = 8.4 Hz, 1H, 4′-H), 6.88–6.85 (m, 2H, Ar-H), 6.67 (t, *J* = 4.7 Hz, 1H, NH), 6.06 (d, *J* = 2.3 Hz, 1H, 8-H), 6.03 (d, *J* = 2.3 Hz, 1H, 6-H), 5.33 (dd, *J* = 12.8, 3.0 Hz, 1H, 2-H), 4.51 (s, 2H, C=OCH_2_O), 4.47 (d, *J* = 5.8 Hz, 2H, Ar-CH_2_N), 3.92 (s, 3H, OCH_3_), 3.80 (s, 3H, OCH_3_), 3.08 (dd, *J* = 17.2, 12.9 Hz, 1H, 3-H), 2.80 (dd, *J* = 17.2, 3.1 Hz, 1H, 3-H). APCI-MS: calculated for [M + Na]^+^ (C_26_H_25_NNaO_8_) requires *m*/*z* 502.1472, found 502.1469.

*2-[5-Hydroxy-2-(3-hydroxy-4-methoxyphenyl)-4-oxochroman-7-yloxy]-N-(4-trifluoro-methylbenzyl)acetamide* (**4f**). White solid (70% yield); ^1^H-NMR (400 MHz, DMSO) δ 12.07 (s, 1H, 5-OH), 9.08 (s, 1H, 3′-OH), 8.77 (t, *J* = 6.1 Hz, 1H, NH), 7.66 (d, *J* = 8.1 Hz, 2H, Ar-H), 7.47 (d, *J* = 8.0 Hz, 2H, Ar-H), 6.94 (m, 2H, 2′-H, 4′-H), 6.88 (dd, *J* = 8.4, 2.0 Hz, 1H, 5′-H), 6.14 (d, *J* = 2.3 Hz, 1H, 8-H), 6.12 (d, *J* = 2.3 Hz, 1H, 6-H), 5.50 (dd, *J* = 12.4, 3.0 Hz, 1H, 2-H), 4.65 (s, 2H, C=OCH_2_O), 4.42 (d, *J* = 6.0 Hz, 2H, Ar-CH_2_N), 3.78 (s, 3H, OCH_3_), 3.27 (dd, *J* = 17.2, 12.5 Hz, 1H, 3-H), 2.77 (dd, *J* = 17.2, 3.1 Hz, 1H, 3-H). APCI-MS: calculated for [M + Na]^+^ (C_26_H_31_F_3_NNaO_7_) requires *m*/*z* 528.1913, found 528.1910.

*N-(2-Chlorobenzyl)-2-[5-hydroxy-2-(3-hydroxy-4-methoxyphenyl)-4-oxochroman-7-yloxy]acetamide* (**4g**). White solid (59% yield); ^1^H-NMR (400 MHz, CDCl_3_) δ 11.97 (s, 1H, 5-OH), 7.34 (td, *J* = 7.4, 1.5 Hz, 1H, Ar-H), 7.31–7.25 (m, 2H, Ar-H), 7.11 (td, *J* = 7.5, 1.1 Hz, 1H, Ar-H), 7.09–7.03 (m, 1H, Ar-H), 7.03 (d, *J* = 2.0 Hz, 1H, 2′-H), 6.92 (dd, *J* = 8.4, 1.9 Hz, 1H, 5′-H), 6.88 (d, *J* = 8.3 Hz, 1H, 4′-H), 6.81 (t, *J* = 5.5 Hz, 1H, NH), 6.08 (d, *J* = 2.4 Hz, 1H, 8-H), 6.05 (d, *J* = 2.4 Hz, 1H, 6-H), 5.33 (dd, *J* = 12.8, 3.0 Hz, 1H, 2-H), 4.59 (d, *J* = 6.1 Hz, 2H, NCH_2_), 4.51 (s, 2H, C=OCH_2_O), 3.92 (s, 3H, OCH_3_), 3.08 (dd, *J* = 17.2, 12.9 Hz, 1H, 3-H), 2.81 (dd, *J* = 17.2, 3.1 Hz, 1H, 3-H). APCI-MS: calculated for [M + Na]^+^ (C_25_H_22_ClNNaO_7_) requires *m*/*z* 506.0978, found 506.0977.

*N-(2-Fluorobenzyl)-2-[5-hydroxy-2-(3-hydroxy-4-methoxyphenyl)-4-oxochroman-7-yloxy]-acetamide* (**4h**). White solid (65% yield); ^1^H-NMR (400 MHz, CDCl_3_) δ 11.97 (s, 1H, 5-OH), 7.37–7.27 (m, 2H, Ar-H), 7.11 (td, *J* = 7.5, 1.1 Hz, 2H, Ar-H), 7.03 (d, *J* = 2.0 Hz, 1H, 2′-H), 6.92 (dd, *J* = 8.3, 2.0 Hz, 1H, 5′-H), 6.88 (d, *J* = 8.3 Hz, 1H, 4′-H), 6.81 (t, *J* = 5.5 Hz, 1H, NH), 6.08 (d, *J* = 2.4 Hz, 1H, 8-H), 6.05 (d, *J* = 2.4 Hz, 1H, 6-H), 5.33 (dd, *J* = 12.8, 3.0 Hz, 1H, 2-H), 4.59 (d, *J* = 6.1 Hz, 2H, NCH_2_), 4.51 (s, 2H, C=OCH_2_O), 3.92 (s, 3H, OCH_3_), 3.08 (dd, *J* = 17.2, 12.9 Hz, 1H, 3-H), 2.81 (dd, *J* = 17.2, 3.1 Hz, 1H, 3-H). APCI-MS: calculated for [M + H]^+^ (C_25_H_23_FNO_7_) requires *m*/*z* 468.1453, found 468.1466.

*2-[5-Hydroxy-2-(3-hydroxy-4-methoxyphenyl)-4-oxochroman-7-yloxy]-N-(2-methoxy-benzyl)acetamide* (**4i**). White solid (64% yield); ^1^H-NMR (400 MHz, DMSO) δ 12.07 (s, 1H, 5-OH), 9.09 (s, 1H, 3′-OH), 8.43 (t, *J* = 5.9 Hz, 1H, NH), 7.26–7.19 (m, 1H, Ar-H), 7.15–7.10 (m, 1H, Ar-H), 6.99–6.92 (m, 3H, 2′-H, Ar-H), 6.87 (m, 2H, 4′-H, 5′-H), 6.12 (d, *J* = 2.3 Hz, 1H, 8-H), 6.11 (d, *J* = 2.3 Hz, 1H, 6-H), 5.49 (dd, *J* = 12.5, 3.0 Hz, 1H, 2-H), 4.64 (s, 2H, C=OCH_2_O), 4.30 (d, *J* = 5.9 Hz, 2H, Ar-CH_2_N), 3.79 (s, 3H, OCH_3_), 3.78 (s, 3H, OCH_3_), 3.27 (dd, *J* = 17.2, 12.6 Hz, 1H, 3-H), 2.76 (dd, *J* = 17.2, 3.1 Hz, 1H, 3-H). APCI-MS: calculated for [M + H]^+^ (C_26_H_26_NO_8_) requires *m*/*z* 480.1653, found 480.1653.

*2-[5-Hydroxy-2-(3-hydroxy-4-methoxyphenyl)-4-oxochroman-7-yloxy]-N-(3-methoxy-benzyl)acetamide* (**4j**). White solid (72% yield); ^1^H-NMR (400 MHz, CDCl_3_) δ 11.97 (s, 1H, 5-OH), 7.28–7.22 (m, 1H, Ar-H), 7.02 (d, *J* = 2.0 Hz, 1H, 2′-H), 6.92 (dd, *J* = 8.3, 2.0 Hz, 1H, 5′-H), 6.88 (d, *J* = 8.2 Hz, 1H, 4′-H), 6.88–6.80 (m, 3H, Ar-H), 6.72 (t, *J* = 5.5 Hz, 1H, NH), 6.07 (d, *J* = 2.4 Hz, 1H, 8-H), 6.05 (d, *J* = 2.4 Hz, 1H, 6-H), 5.33 (dd, *J* = 12.8, 3.0 Hz, 1H, 2-H), 4.54 (s, 2H, C=OCH_2_O), 4.51 (d, *J* = 5.9 Hz, 2H, NCH_2_), 3.92 (s, 3H, OCH_3_), 3.79 (s, 3H, OCH_3_), 3.08 (dd, *J* = 17.2, 12.9 Hz, 1H, 3-H), 2.81 (dd, *J* = 17.2, 3.1 Hz, 1H, 3-H). APCI-MS: calculated for [M + Na]^+^ (C_26_H_25_NNaO_8_) requires *m*/*z* 502.1472, found 502.1473.

*2-[5-Hydroxy-2-(3-hydroxy-4-methoxyphenyl)-4-oxochroman-7-yloxy]-N-phenethyl-acetamide* (**4k**). White solid (52% yield); ^1^H-NMR (400 MHz, CDCl_3_) δ 11.99 (s, 1H, 5-OH), 7.32–7.27 (m, 2H, Ar-H), 7.25–7.21 (m, 1H, Ar-H), 7.19–7.15 (m, 2H, Ar-H), 7.04 (d, *J* = 2.0 Hz, 1H, 2′-H), 6.92 (dd, *J* = 8.4, 2.0 Hz, 1H, 5′-H), 6.89 (d, *J* = 8.3 Hz, 1H, 4′-H), 6.45 (t, *J* = 5.3 Hz, 1H, NH), 6.02 (d, *J* = 2.4 Hz, 1H, 8-H), 5.99 (d, *J* = 2.4 Hz, 1H, 6-H), 5.34 (dd, *J* = 12.8, 3.0 Hz, 1H, 2-H), 4.45 (s, 2H, C=OCH_2_N), 3.92 (s, 3H, OCH_3_), 3.60 (dd, *J* = 13.0, 6.9 Hz, 2H, NCH_2_), 3.09 (dd, *J* = 17.2, 12.8 Hz, 1H, 3-H), 2.88–2.78 (m, 3H, Ar-CH_2_, 3-H). APCI-MS: calculated for [M + Na]^+^ (C_26_H_25_NNaO_7_) requires *m*/*z* 486.1523, found 486.1525.

*2-[5-Hydroxy-2-(3-hydroxy-4-methoxyphenyl)-4-oxochroman-7-yloxy]-N-[2-(4-methoxy-phenyl)ethyl]acetamide* (**4l**). White solid (57% yield); ^1^H-NMR (400 MHz, CDCl_3_) δ 11.99 (s, 1H, 5-OH), 7.10–7.05 (m, 2H, Ar-H), 7.04 (d, *J* = 2.0 Hz, 1H, 2′-H), 6.93 (dd, *J* = 8.3, 2.0 Hz, 1H, 5′-H), 6.89 (d, *J* = 8.3 Hz, 1H, 4′-H), 6.86–6.81 (m, 2H, Ar-H), 6.41 (t, *J* = 5.3 Hz, 1H, NH), 6.01 (d, *J* = 2.3 Hz, 1H, 8-H), 5.99 (d, *J* = 2.3 Hz, 1H, 8-H), 5.35 (dd, *J* = 12.8, 3.0 Hz, 1H, 2-H), 4.45 (s, 2H, C=OCH_2_O), 3.92 (s, 3H, OCH_3_), 3.78 (s, 3H, OCH_3_), 3.56 (q, *J* = 6.8 Hz, 2H, NCH_2_), 3.10 (dd, *J* = 17.2, 12.8 Hz, 1H, 3-H), 2.80 (dt, *J* = 10.7, 5.0 Hz, 3H, Ar-CH_2_, 3-H). APCI-MS: calculated for [M + Na]^+^ (C_27_H_27_NNaO_8_) requires *m*/*z* 516.1629, found 516.1628.

*N-[2-(4-Chlorophenyl)ethyl]-2-[5-hydroxy-2-(3-hydroxy-4-methoxyphenyl)-4-oxo-chroman-7-yloxy]acetamide* (**4m**). White solid (66% yield); ^1^H-NMR (400 MHz, CDCl_3_) δ 12.00 (s, 1H, 5-OH), 7.26–7.23 (m, 2H, Ar-H), 7.12–7.07 (m, 2H, Ar-H), 7.04 (d, *J* = 2.0 Hz, 1H, 2′-H), 6.93 (dd, *J* = 8.4, 2.0 Hz, 2H. 5′-H), 6.89 (d, *J* = 8.3 Hz, 1H, 4′-H), 6.41 (t, *J* = 5.6 Hz, 1H, NH), 6.02 (d, *J* = 2.4 Hz, 1H, 8-H), 6.00 (d, *J* = 2.4 Hz, 1H, 6-H), 5.35 (dd, *J* = 12.8, 3.1 Hz, 1H, 2-H), 4.46 (s, 2H, C=OCH_2_O), 3.92 (s, 3H, OCH_3_), 3.58 (q, *J* = 6.6 Hz, 2H, NCH_2_), 3.10 (dd, *J* = 17.2, 12.8 Hz, 1H, 3-H), 2.86–2.78 (m, 3H, Ar-CH_2_, 3-H). APCI-MS: calculated for [M + Na]^+^ (C_26_H_24_ClNNaO_7_) requires *m*/*z* 520.1134, found 520.1136.

*N-[2-(4-Bromophenyl)ethyl]-2-[5-hydroxy-2-(3-hydroxy-4-methoxyphenyl)-4-oxo-chroman-7-yloxy]acetamide* (**4n**). White solid (70% yield); ^1^H-NMR (400 MHz, CDCl_3_) δ 12.00 (s, 1H, 5-OH), 7.43–7.38 (m, 2H, Ar-H), 7.07–7.01 (m, 3H, Ar-H, 2′-H), 6.93 (dd, *J* = 8.4, 2.0 Hz, 1H, 5′-H), 6.89 (d, *J* = 8.3 Hz, 1H, 4′-H), 6.41 (t, *J* = 5.5 Hz, 1H, NH), 6.03 (d, *J* = 2.4 Hz, 1H, 8-H), 6.00 (d, *J* = 2.4 Hz, 1H, 6-H), 5.35 (dd, *J* = 12.8, 3.1 Hz, 1H, 2-H), 4.46 (s, 2H, C=OCH_2_O), 3.92 (s, 3H, OCH_3_), 3.57 (q, *J* = 6.3 Hz, 2H, NCH_2_), 3.10 (dd, *J* = 17.2, 12.8 Hz, 1H, 3-H), 2.87–2.78 (m, 3H, Ar-CH_2_, 3-H). APCI-MS: calculated for [M + Na]^+^ (C_26_H_24_BrNNaO_7_) requires *m*/*z* 564.0628, found 564.0627.

*N-[2-(4-Fluorophenyl)ethyl]-2-[5-hydroxy-2-(3-hydroxy-4-methoxyphenyl)-4-oxochroman-7-yloxy]-acetamide* (**4o**). White solid (40% yield); ^1^H-NMR (400 MHz, CDCl_3_) δ 12.00 (s, 1H, 5-OH), 7.15–7.09 (m, 2H, Ar-H), 7.04 (d, *J* = 2.0 Hz, 1H, 2′-H), 6.97 (ddd, *J* = 10.8, 5.8, 2.5 Hz, 2H, Ar-H), 6.93 (dd, *J* = 8.5, 2.0 Hz, 1H, 5′-H), 6.89 (d, *J* = 8.3 Hz, 1H, 4′-H), 6.42 (t, *J* = 5.5 Hz, 1H, NH), 6.02 (d, *J* = 2.4 Hz, 1H, 8-H), 5.99 (d, *J* = 2.4 Hz, 1H, 6-H), 5.34 (dd, *J* = 12.8, 3.1 Hz, 1H, 2-H), 4.45 (s, 2H, C=OCH_2_O), 3.92 (s, 3H, OCH_3_), 3.57 (dd, *J* = 13.2, 6.9 Hz, 2H, NH), 3.10 (dd, *J* = 17.2, 12.8 Hz, 1H, 3H), 2.85–2.79 (m, 3H, Ar-CH_2_, 3-H). APCI-MS: calculated for [M + H]^+^ (C_26_H_25_FNO_7_) requires *m*/*z* 482.1610, found 482.1612.

*N-[2-(2-Chlorophenyl)ethyl]-2-[5-hydroxy-2-(3-hydroxy-4-methoxyphenyl)-4-oxo-chroman-7-yloxy]acetamide* (**4p**). White solid (55% yield); ^1^H-NMR (400 MHz, CDCl_3_) δ 11.99 (s, 1H, 5-OH), 7.38–7.33 (m, 1H, Ar-H), 7.21–7.16 (m, 3H, Ar-H), 7.04 (d, *J* = 2.0 Hz, 1H, 2′-H), 6.92 (dd, *J* = 8.4, 2.0 Hz, 1H, 5′-H), 6.89 (d, *J* = 8.3 Hz, 1H, 4′-H), 6.51 (t, *J* = 5.5 Hz, 1H, NH), 6.03 (d, *J* = 2.4 Hz, 1H, 8-H), 6.01 (d, *J* = 2.4 Hz, 1H, 6-H), 5.34 (dd, *J* = 12.8, 3.1 Hz, 1H, 2-H), 4.45 (s, 2H, C=OCH_2_O), 3.92 (s, 3H, OCH_3_), 3.63 (dd, *J* = 12.9, 6.8 Hz, 2H, NCH_2_), 3.09 (dd, *J* = 17.2, 12.8 Hz, 1H, 3-H), 3.01 (t, *J* = 6.9 Hz, 2H, Ar-CH_2_), 2.82 (dd, *J* = 17.2, 3.1 Hz, 1H, 3-H). APCI-MS: calculated for [M + Na]^+^ (C_26_H_24_ClNNaO_7_) requires *m*/*z* 520.1134, found 520.1135.

*N-[2-(2-Fluorophenyl)ethyl]-2-[5-hydroxy-2-(3-hydroxy-4-methoxyphenyl)-4-oxochroman-7-yloxy]-acetamide* (**4q**). White solid(60% yield); ^1^H-NMR (400 MHz, DMSO) δ 12.07 (s, 1H, 5-OH), 9.09 (s, 1H, 3′-OH), 8.22 (t, *J* = 5.7 Hz, 1H, NH), 7.33–7.20 (m, 3H, Ar-H), 7.19–7.04 (m, 2H, Ar-H, 2′-OH), 6.98–6.86 (m, 2H, 4′-H, 5′-H), 6.09–6.07 (m, 2H, 6-H, 8-H), 5.49 (dd, *J* = 12.4, 2.9 Hz, 1H, 2-H), 4.52 (s, 2H, C=OCH_2_O), 3.78 (s, 3H, OCH_3_), 3.35 (dd, *J* = 13.0, 6.8 Hz, 2H, NCH_2_), 3.26 (dd, *J* = 17.2, 12.3 Hz, 1H, 3-H), 2.81–2.73 (dd, *J* = 17.0, 3.0 Hz, 1H, 3-H). APCI-MS: calculated for [M + Na]^+^ (C_26_H_2**4f**_NNaO_7_) requires *m*/*z* 504.1429, found 504.1434.

*N-[2-(3-Chlorophenyl)ethyl]-2-[5-hydroxy-2-(3-hydroxy-4-methoxyphenyl)-4-oxo-chroman-7-yloxy]acetamide* (**4r**). White solid (62% yield); ^1^H-NMR (400 MHz, CDCl_3_) δ 11.99 (s, 1H, 5-OH), 7.25–7.13 (m, 2H, Ar-H), 7.10–7.00 (m, 3H, Ar-H, 2′-H), 6.93 (dd, *J* = 8.3, 2.0 Hz, 1H, 5′-H), 6.89 (d, *J* = 8.3 Hz, 1H, 4′-H), 6.52 (t, *J* = 5.3 Hz, 1H, NH), 6.03 (d, *J* = 2.3 Hz, 1H, 8-H), 6.01 (d, *J* = 2.3 Hz, 1H, 6-H), 5.34 (dd, *J* = 12.8, 3.0 Hz, 1H, 2-H), 4.45 (s, 2H, C=OCH_2_O), 3.92 (s, 3H, OCH_3_), 3.61 (dd, *J* = 12.9, 6.7 Hz, 2H, NCH_2_), 3.09 (dd, *J* = 17.2, 12.8 Hz, 1H, 3-H), 2.91 (t, *J* = 6.8 Hz, 1H, Ar-CH_2_), 2.82 (dd, *J* = 17.2, 3.1 Hz, 1H, 3-H). APCI-MS: calculated for [M + Na]^+^ (C_26_H_24_ClNNaO_7_) requires *m*/*z* 520.1134, found520.1131.

*2-[5-Hydroxy-2-(3-hydroxy-4-methoxyphenyl)-4-oxochroman-7-yloxy]-N-pyridin-4-ylmethylacetamide* (**4s**). White solid (68% yield); ^1^H-NMR (400 MHz, DMSO) δ 12.07 (s, 1H, 5-OH), 9.09 (s, 1H, 3′-OH), 8.75 (t, *J* = 6.1 Hz, 1H, NH), 8.47 (dd, *J* = 4.4, 1.6 Hz, 2H, Py-H), 7.24 (d, *J* = 6.0 Hz, 2H, Py-H), 6.96–6.92 (m, 2H, 2′-H, 4′-H), 6.91–6.86 (m, 1H, 5′-H), 6.14 (d, *J* = 2.3 Hz, 1H, 8-H), 6.13 (d, *J* = 2.3 Hz, 1H, 6-H), 5.50 (dd, *J* = 12.4, 3.0 Hz, 1H, 2-H), 4.68 (s, 2H, C=OCH_2_O), 4.36 (d, *J* = 6.1 Hz, 2H, Py-CH_2_), 3.78 (s, 3H, OCH_3_),3.27 (dd, *J* = 17.0, 12.2 Hz, 1H, 3-H), 2.77 (dd, *J* = 17.2, 3.1 Hz, 1H, 3-H). APCI-MS: calculated for [M + H]^+^ (C_24_H_23_N_2_O_7_) requires *m*/*z* 451.1500, found 451.1501.

*N-Furan-2-ylmethyl-2-[5-hydroxy-2-(3-hydroxy-4-methoxyphenyl)-4-oxochroman-7-yloxy]acetamide* (**4t**). White solid (56% yield); ^1^H-NMR (400 MHz, CDCl_3_) δ 11.98 (s, 1H, 5-oh), 7.37 (dd, *J* = 1.8, 0.8 Hz, 1H, Fu-H), 7.03 (d, *J* = 2.0 Hz, 1H, 2′-H), 6.92 (dd, *J* = 8.4, 2.0 Hz, 1H, 5′-H), 6.88 (d, *J* = 8.3 Hz, 1H, 4′-H), 6.76 (t, *J* = 4.6 Hz, 1H, NH), 6.33 (dd, *J* = 3.2, 1.9 Hz, 1H, Fu-H), 6.26 (dd, *J* = 3.2, 0.6 Hz, 1H, Fu-H), 6.08 (d, *J* = 2.4 Hz, 1H, 8-H), 6.05 (d, *J* = 2.4 Hz, 1H, 6-H), 5.34 (dd, *J* = 12.8, 3.0 Hz, 1H, 2-H), 4.53 (d, *J* = 5.8 Hz, 2H, NCH_2_), 4.51 (s, 2H, C=OCH_2_O), 3.92 (s, 3H, OCH_3_), 3.08 (dd, *J* = 17.2, 12.8 Hz, 1H, 3-H), 2.81 (dd, *J* = 17.2, 3.1 Hz, 1H, 3-H). APCI-MS: calculated for [M + Na]^+^ (C_23_H_21_NNaO_8_) requires *m*/*z* 462.1159, found 462.1158.

### 3.3. In Vitro Inhibition Studies on AChE and BuChE

Acetylcholinesterase (AChE, E.C. 3.1.1.7, obtained from electric eel) was purchased from Sigma-Aldrich (St. Louis, MO, USA, product code: C3389-2KU). Butyrylcholinesterase (BuChE, E.C. 3.1.1.8, obtained from equine serum) was purchased from Aladdin (Shanghai, China, product code: B128570-2KU). 5,5′-dithiobis-(2-nitrobenzoic acid) (Ellman’s reagent DTNB), acetylthiocholine chloride (ATC), butylthiocholine chloride (BTC), donepezil hydrochloride and tacrine hydrochloride were purchased from Sigma-Aldrich. The compounds were dissolved in a minimum volume of DMSO (1%) and then diluted in 0.1 M KH_2_PO_4_/K_2_HPO_4_ buffer (pH 8.0) to provide a final concentration range.

All the methods were under 0.1 M KH_2_PO_4_/K_2_HPO_4_ buffer (pH 8.0), using a Shimadzu UV-2450 Spectrophotometer. Enzyme solutions were prepared to give 2.0 units/ mL in 2 mL aliquots. The assay medium contained phosphate buffer, pH 8.0 (1 mL), 50 μL of 0.01 M DTNB, 10 μL of enzyme, and 50 μL of 0.01 M substrate (ATC). The substrate was added to the assay medium containing enzyme, buffer, and DTNB with inhibitor after 15 min of incubation time. The activity was determined by measuring the increase in absorbance at 412 nm at 1 min intervals at 37 °C. In vitro BuChE assay used the similar method described above [31]. Each concentration was assayed in triplicate.

### 3.4. Kinetic Characterization of AChE Inhibition

Three different concentrations (2, 4 and 8 nM) of substrate were mixed in the 1 mL 0.1 M KH_2_PO_4_/K_2_HPO_4_ buffer (pH 8.0), containing 10 μL AChE, 50 μL of DTNB, and 50 μL substrate. The test compound **4f** was added into the assay solution and pre-incubated with the enzyme at 37 °C for 15 min, followed by the addition of substrate. Kinetic characterization of the hydrolysis of ATC-catalyzed by AChE was done spectrometrically at 412 nm. A parallel control with no inhibitor in the mixture, allowed adjusting activities to be measured at various times. Kinetic characterization of BuChE assay used the similar method described above.

### 3.5. Molecular Modeling

The crystal structure of the torpedo AChE (code ID: 1ACJ) was obtained in the Protein Data Bank after eliminating the inhibitor and water molecules. Docking studies were carried out using the AutoDock4.0 (The Scripps Research Institute, San Diego, CA, USA), we adopted the hybrid Lamarckian genetic algorithm as the searching algorithm to provide the compound with full flexibility. The resulting enzyme structure was used as an input for the AutoGrid program. AutoGrid performed precalculated atomic affinity grid maps for each atom type in the ligand plus a separate desolvation map, and a separate desolvation map present in the substrate molecule. All maps were calculated with 0.375 Å spacing between grid points. The center of the grid box was placed at the bottom of the active site gorge (AChE 2.781 64.383 67.971). The dimensions of the active site box were set at 50 × 50 × 50 Å.

### 3.6. Measurement of the Anti-Oxidation Activity

The anti-oxidation activity was tested by using the oxygen radical absorbance capacity-fluorescein (ORAC-FL) assay [25,32]. All the ORAC-assays measure antioxidant scavenging activity against peroxyl radical induced by 2,2′-azobis (2-amidinopropane) dihydrochloride (AAPH) at 37 °C.

The reaction was conducted with 75 mM phosphate buffer (pH 7.4) and the final reaction mixture was 200 μL. Fluorescein (120 μL, 300 nM final concentration) and antioxidant (20 μL) were placed in the wells of a black 96-well plate, and the mixture was incubated for 15 min at 37 °C. Then AAPH solution (60 μL, 12 mM final concentration) was added rapidly. The plate was immediately placed into a Varioskan Flash Multimode Reader (Thermo Scientific, Waltham, MA, USA) and the fluorescence was measured every 60 seconds for 120 min with excitation at 485 nm and emission at 535 nm. 6-hydroxy-2,5,7,8-tetramethylchroman-2-carboxylic acid (Trolox) was used as a standard (1–5 μM, final concentration). A blank (FL + AAPH) using phosphate buffer instead of antioxidant and Trolox calibration were carried out in each assay. The samples were measured at different concentrations (1–5 μM), and at least three independent assays were performed for each sample. Fluorescence measurements were normalized based on the curve of the blank (without antioxidant). Antioxidant curves (fluorescence versus time) were normalized to the curve of the blank in the same assay, and then the area under the fluorescence decay curve (AUC) was calculated. The net AUC of a sample was obtained by subtracting the AUC from the blank. ORAC-FL values were expressed as Trolox equivalents by using the standard curve calculated for each sample, where the ORAC-FL value of Trolox was taken as one, indicating the antioxidant potency of the test compounds. The equations of the results calculations are as follows:

(1) AUC = 1 +$\;\sum_{F0}^{Fi}Fi/F0$ (*F*0 is the initial fluorescence at 0 min, and it is the fluorescence at time I);
(2) The ORAC values were calculated as following: [(AUC_sample_ − AUC_blank_)/(AUC_Trolox_ − AUC_blank_)]/[(concentration of Trolox/concentration of sample)], and expressed as Trolox equivalents by using the standard curve calculated for each assay. Final result were in μM of Trolox equivalent/μM of pure compound.

### 3.7. Inhibition Experiments of Self-Mediated Aβ (1–42) Aggregation

A thioflavin T based fluorometric assay was performed to investigate the self-mediated Aβ (1–42) aggregation [33]. Aβ (1–42) peptide (Sigma-Aldrich) was dissolved in phosphate buffer (0.01 M, pH 7.40) to obtain a 20 μM solution. Compounds were firstly dissolved in DMSO to obtain a 10 mM solution. Inhibitors and the final concentration of Aβ (1–42) were 20 μM. After incubated in 37 °C for 48 h, thioflavin-T (5 μM in 50 mM glycine-NaOH buffer, pH 8.00) was added. Fluorescence was measured at 450 nm (λ_ex_) and 485 nm (λ_em_). Each inhibitor was run in triplicate. The fluorescence intensities were recorded, and the percent inhibition on aggregation was calculated by the following expression: (1 − I_Fi_/I_Fc_) × 100% in which I_Fi_ and I_Fc_ were the fluorescence intensities obtained for absorbance in the presence and absence of inhibitors, respectively, after subtracting the fluorescence of respective blanks.

### 3.8. Protective Effect Against H_2_O_2_-Induced Cell Death in PC12Neurons

PC12 cells were cultured in DMEM media with 10% fetal calf serum (FCS, Hyclone) containing 100 units/mL penicillin and 100 mg/mL streptomycin under 5% CO_2_ at 37 °C. To produce oxidative stress, H_2_O_2_ was freshly prepared from a 30% stock solution prior to each experiment. PC12 cells were plated in cell culture plates at a density of 10^5^ cells per well. In our study, 0.2 mM H_2_O_2_ were treated with PC12 cells for 24 h in the injury module, while the compound group cells were pretreated with different concentrations (1, 2.5 and 5 μM) of the compound **4f** for 2 h. The control group cells were treated with the same medium without H_2_O_2_ or compound **4f**. The cell viability of PC12 cells was evaluated using MTT assay. After, PC12 cells were cultured with 5 mg/mL MTT and incubated for 4 h at 37 °C. Then the cells were lysed in dimethyl sulfoxide DMSO (200 mL). The absorbance was detected at 570 nm by a microplate reader (Bio-Tek). PC12 cells were cultured without **4f** or H_2_O_2_ as control group and the results were expressed by percentage of control.

### 3.9. Cellular Toxicity

The SH-SY5Y cell line was seeded on 96-well plates at a density of 10^4^ cells/well. After overnight incubation, the cells were treated with various concentrations (1, 10 and 100 μM) of hesperetin derivatives for 48 h. Subsequently, 10 μL of 5 mg/mL MTT solution was added to each well and further incubated for 4 h at 37 °C. The solution was carefully removed, and each well was treated with DMSO (200 μL for each well). The optical density of the wells was then read on the microplate reader at 570 nm. All the tested compounds were performed in triplicate.

## 4. Conclusions

In summary, a series of hesperetin derivativeswere designed and synthesized on the basis of the structural characteristics of the AChE dual-site inhibitors as multifunctional anti-Alzheimer’s Disease agent. Our results showed that these derivatives have high inhibitory potency and selectivity for AChE. Compound **4f** showed the highest inhibitory activity for AChE (IC_50_ = 9.37 nM, selectivity index = 305). The inhibition kinetic analysis and molecular modeling study indicated that compound **4f** binds with both the CAS and PAS of the AChE. Moreover, these hesperetin derivatives have significant peroxyl radical absorbance capacity and anti-Aβ aggregation activity. In addition, compound **4f** also shows neuroprotective activity against the H_2_O_2_-induced cell death in PC12 cells. The results show the potential of the prototype compound **4f** as a multiple potency anti-AD agent for further developments.

## Figures and Tables

**Table 1:** In vitro inhibition of AChE, BuChE and oxygen radical absorbance capacity (ORAC, Trolox equivalents) of the synthesized hesperetin derivatives.

Compounds	AchE Inhibition ^a^, (IC_50_) nM	BuChE Inhibition ^b^, (IC_50_) nM	Selective Index ^c^	ORAC ^d^
Hesperetin	1023 ± 37	2897 ± 67	3	5.1
**4a**	25.51 ± 1.32	3400 ± 56	133	3.2
**4b**	13.55 ± 0.78	3502 ± 66	258	2.7
**4c**	12.44 ± 0.81	2078 ± 76	167	2.9
**4d**	15.71 ± 0.95	2133 ± 64	136	2.6
**4e**	17.88 ± 054	3208 ± 92	179	2.8
**4f**	9.37 ± 0.87	2862 ± 45	305	3.0
**4g**	14.03 ± 0.95	1634 ± 78	116	3.1
**4h**	15.23 ± 2.03	2803 ± 39	184	2.5
**4i**	19.23 ± 0.66	1985 ± 134	103	2.7
**4j**	22.33 ± 1.54	2456 ± 83	110	3.1
**4k**	11.87 ± 1.12	3013 ± 74	254	2.7
**4l**	19.52 ± 0.77	2790 ± 53	143	2.9
**4m**	15.67 ± 0.85	3354 ± 66	214	2.6
**4n**	17.22 ± 0.96	1986 ± 51	115	2.4
**4o**	13.52 ± 1.44	2255 ± 81	167	3.1
**4p**	17.22 ± 0.45	3469 ± 67	201	2.7
**4q**	20.22 ± 1.88	2235 ± 77	111	2.5
**4r**	16.11 ± 0.79	3025 ± 84	188	2.8
**4s**	27.32 ± 0.99	1846 ± 55	68	2.4
**4t**	30.65 ± 4.50	2355 ± 104	77	2.6
Tacrine	203.5 ± 3.25	30.50 ± 1.49	0.15	<0.1
Donepezil	17.57 ± 1.01	5634 ± 76	320.7	<0.1
Curcumin	—	—	—	2.7

^a^ AChE from electric eel; IC_50_, 50% inhibitor concentration (means ± SEM of three experiments); ^b^ BuChE from equine serum; IC_50_, 50% inhibitor concentration (means ± SEM of three experiments); ^c^ Selectivity Index = IC_50_ (BuChE)/IC_50_ (AChE); ^d^ Results are expressed as μM of Trolox equivalent/μMof tested compound.

**Table 2:** Inhibition for SH-SY5Y of the compounds and curcumin at different concentrations.

Compounds	Cell Viability ^a^ (%, 1 μM)	Cell Viability (%, 10 μM)	Cell Viability (%, 50 μM)	Cell Viability (%, 100 μM)
Hesperetin	99.01 ± 5.87	93.03 ± 4.33	85.23 ± 3.43	67.34 ± 5.21
**4a**	98.23 ± 8.02	91.56 ± 5.67	81.43 ± 5.23	63.21 ± 4.23
**4b**	102.52 ± 4.56	95.87 ± 3.98	83.66 ± 4.12	65.41 ± 5.33
**4c**	98.52 ± 4.98	97.54 ± 3.76	83.78 ± 2.32	58.75 ± 3.97
**4d**	99.87 ± 5.76	99.45 ± 6.23	86.23 ± 5.14	70.02 ± 4.78
**4e**	104.91 ± 3.87	98.32 ± 5.27	87.13 ± 6.37	66.54 ± 4.95
**4f**	100.23 ± 4.76	95.77 ± 7.31	83.29 ± 5.44	62.32 ± 5.78
**4g**	97.85 ± 6.83	95.65 ± 5.67	79.33 ± 4.33	70.81 ± 5.32
**4h**	100.02 ± 6.52	98.75 ± 5.38	75.79 ± 3.83	60.65 ± 4.27
**4i**	97.85 ± 6.32	96.65 ± 5.21	74.95 ± 4.12	55.05 ± 5.69
**4j**	103.21 ± 6.47	98.21 ± 7.01	84.24 ± 3.20	70.80 ± 5.36
**4k**	98.88 ± 6.30	96.55 ± 2.98	80.98 ± 3.22	68.54 ± 4.92
**4l**	98.87 ± 4.07	94.54 ± 5.32	78.53 ± 3.21	63.21 ± 5.04
**4m**	102.10 ± 6.65	97.31 ± 5.21	81.51 ± 4.01	64.52 ± 4.32
**4n**	99.76 ± 3.89	96.54 ± 4.24	78.79 ± 5.21	55.61 ± 5.34
**4o**	100.54 ± 6.31	97.23 ± 5.09	81.67 ± 3.52	59.67 ± 6.14
**4p**	99.32 ± 4.34	94.44 ± 3.90	83.08 ± 4.27	64.45 ± 4.82
**4q**	98.96 ± 5.28	92.01 ± 6.41	85.62 ± 3.95	66.54 ± 3.81
**4r**	98.87 ± 5.2	95.81 ± 6.29	83.78 ± 4.24	62.19 ± 4.18
**4s**	100.74 ± 6.17	97.54 ± 5.36	78.65 ± 3.46	59.64 ± 3.61
**4t**	99.87 ± 4.52	97.54 ± 3.65	81.80 ± 4.06	61.23 ± 5.12
Curcumin	95.34 ± 5.21	90.87 ± 4.31	82.07 ± 3.07	71.83 ± 4.21

^a^ Cell viability: Data are expressed as percent of positive control. Data show mean ± SD for three independent experiments.

## Supplementary Materials

Supplementary File 1
