# Supplementary material for: Design, Synthesis and Evaluation of Hesperetin Derivatives as Potential Multifunctional Anti-Alzheimer Agents
Source: Molecules. 2017 Jun 26;22(7):1067. doi: 10.3390/molecules22071067 (PMC6152180; doi:10.3390/molecules22071067)
Supplement: Supplementary File 1 [file molecules-22-01067-s001.pdf]

We certify that the following article

Design, Synthesis, and Evaluation of Hesperetin Derivatives as Potential Multifunctional anti-Alzheimer Agents

Jun Li

has undergone English language editing by MDPI. The text has been checked for correct use of grammar and common technical terms, and edited to a level suitable for reporting research in a scholarly journal. MDPI uses experienced, native English speaking editors. Full details of the editing service can be found at ► www.mdpi.com/authors/english.

MDPI Multidisciplinary Digital Publishing Institute

Basel, June 2017

Martyn Rittman, Ph.D.
English Editing Manager
